# Green synthesis of iron oxide nanoparticle using *Carica papaya* leaf extract: application for photocatalytic degradation of remazol yellow RR dye and antibacterial activity

**DOI:** 10.1016/j.heliyon.2020.e04603

**Published:** 2020-08-04

**Authors:** Md. Shakhawat Hossen Bhuiyan, Muhammed Yusuf Miah, Shujit Chandra Paul, Tutun Das Aka, Otun Saha, Md. Mizanur Rahaman, Md. Jahidul Islam Sharif, Ommay Habiba, Md. Ashaduzzaman

**Affiliations:** a Department of Applied Chemistry and Chemical Engineering, Noakhali Science and Technology University, Sonapur 3814, Noakhali, Bangladesh; b Department of Pharmacy, Noakhali Science and Technology University, Sonapur 3814, Noakhali, Bangladesh; c Department of Pharmacy, Atish Dipankar University of Science and Technology, Uttara, Dhaka 1230, Bangladesh; d Department of Microbiology, University of Dhaka, Dhaka 1000, Bangladesh; e Department of Applied Chemistry and Chemical Engineering, University of Dhaka, Dhaka 1000, Bangladesh

**Keywords:** Materials science, Materials chemistry, Nanotechnology, Iron oxide nanoparticles, *Carica papaya*, Photocatalytic activity, Remazol yellow RR, Antibacterial activity, Cytotoxicity

## Abstract

Synthesis of iron oxide nanoparticles by the recently developed green approach is extremely promising because of its non-toxicity and environmentally friendly behavior. In this study, nano scaled iron oxide particles (α-Fe_2_O_3_) were synthesized from hexahydrate ferric chloride (FeCl_3_.6H_2_O) with the addition of papaya (*Carica papaya*) leaf extract under atmospheric conditions. The synthesis of iron oxide nanoparticles was confirmed by systematic characterization using FTIR, XRD, FESEM, EDX and TGA studies. The removal efficiency of remazol yellow RR dye with the synthesized iron oxide nanoparticles as a photocatalyst was determined along with emphasizing on the parameters of catalyst dosage, initial dye concentration and pH. Increasing the dose of iron oxide nanoparticles enhanced the decolorization of the dyes and a maximum 76.6% dye degradation was occurred at pH 2 after 6 h at a catalyst dose of 0.8 g/L. Unit removal capacity of the photocatalyst was found to be 340 mg/g at dye concentration of 70 ppm and at a catalyst dose of 0.4 g/L. The synthesized nanoparticles showed moderate antibacterial activity against *Klebsiella* spp.*, E.Coli, Pseudomonas* spp.*, S.aureus* bacterial strains. Although the cytotoxic effect of nanoparticles against Hela, BHK-21 and Vero cell line was found to be toxic at maximum doses but it can be considered for tumor cell damage because it showed excellent activity against the Hela and BHK-21 cell lines.

## 1 Introduction

Quality of surface water is getting deteriorated day by day due to the release of various industrial effluents like dye in recent years. More than 10,000 dyes are now being used in various industries and most of them are used in textile dying purposes. It is estimated that about 15% of the total textile dyes are getting released into the surroundings as effluents and are doing harm to our environments directly or indirectly (Lachheb et al., 2002). Most of the dyes are even entering our life cycle through water, and animals and causing skin irritation, respiratory diseases, and even cancer development (Khan and Malik, 2014; Immich et al., 2009). Remazol yellow RR, remazol red RR, and remazol blue RR dye-different types of reactive azo dyes, are widely being used for coloring the textile fibers, yarns, fabrics, etc. Various kinds of toxicities have been reported for such types of remazol dyes like teratogenicity in frog embryos, enzymic degradation metabolites toxicity, genotoxicity, carcinogenicity, and phytotoxicity (Birhanli and Ozmen, 2005; Silva et al., 2013; Jadhav et al., 2011).

Purification of textile waste effluents particularly dye is a crucial demand for the sake of our mankind, and environment as well. Various kinds of methods like biological methods, filtration, adsorption, sedimentation, ion-exchange, UV treatment, ozonation, etc. are available for purification textile dyes (Hassan and Carr, 2018; Bhatia et al., 2017; Katheresan et al., 2018; Yagub et al., 2014; Robinson et al., 2001; Lau and Ismail, 2009). But most of them are very costly, unavailable and ineffective in some cases. Currently, photodegradation is attracting the scientist to be used for such purposes because this process possesses high efficiency and feasibility as compared to the other traditional methods (López Cisneros et al., 2002). However, these advantages cannot be gained properly without a photocatalyst having high surface area, stability, photocatalytic activity and biocompatibility. Only nanomaterials based photocatalyst found to be possessed such properties and hence are now considering for degradation of textile dyes proficiently. Various types of metal (Ag, Au) and metal oxide (ZnO, TiO_2_) based nanoparticles (NPs) have been studied for utilizing as a photocatalyst for the degradation of textile dyes (Hamaloğlu et al., 2017; Ahammed et al., 2020). Among various types nanomaterials, iron oxide nanoparticles (FO NPs) have excellent catalytic and reductive properties to be used for wastewater treatment and it has the advantage of the ease of separation as compared to the other nanomaterials requiring highly expensive centrifugation for separation (Oliveira et al., 2002). FO NPs are usually used for wide range applications from removal heavy metals, dyes, antibiotics from water sources to the biomedical field like site-specific drug delivery and damaging tumor cell (Vasantharaj et al., 2019; Devi et al., 2019). Again, iron-based nanoparticles found to be effective against various pathogenic bacterial strains and fungi effectively as they can produce highly reactive oxygen species (ROS) (Muthukumar et al., 2019).

There are various available methods for synthesis of nanomaterials like sol-gel method, chemical reduction method, co-precipitation, hydrothermal synthesis etc (Ahmed, 2015). The chemicals used in these methods are considered as harmful for the environment. Therefore, in the recent times green synthesis of nanomaterials has gained importance due to its low cost, simplicity and environmentally friendly nature (Kumar et al., 2014). Green synthesis of nanoparticles utilizes plant extract as both reducing and capping agent eliminating the necessity of harmful reducing agents (Peralta-Videa et al., 2016). Plant extract contains various phytochemicals such as polyphenols, flavonoids, terpenoids, phenolic acids, which are responsible for the reduction and formation of stabilized nanoparticles (Izadiyan et al., 2020). Magnetic nanoparticle synthesized by green methods are non-toxic as compared to nanoparticles synthesized using sodium borohydride (Mahdavi et al., 2013). Recently, several studies were carried out for green synthesis of iron-based nanoparticles from various plants parts like fruit extract of *Cynometra ramiflora,* rind of *Persea americana*, seeds extract of *Punica granatum*, flower extract of *Avicennia marina, etc. for the degradation of various textile dyes* (Bishnoi et al., 2018; Kamaraj et al., 2019; Bibi et al., 2019; Karpagavinayagam and Vedhi, 2019).

The goal of this study was to synthesize the iron oxide (α-Fe_2_O_3_) NPs by using papaya plant leaf extract (*Carica papaya*) as reducing/stabilizing agent and studying its photocatalytic efficiency for the degradation of reactive azo dye (remazol yellow RR), antibacterial activity against bacterial strains and its probable in-vitro cytotoxicity.

## 2 Materials and methods

### 2.1 Chemicals

Analytical grade ferric chloride hexahydrate (FeCl_3_.6H_2_O), sodium hydroxide pellets (NaOH) were purchased from Merck, India. All chemicals are used without further purification. Remazol yellow RR dye was collected from local textile of Bangladesh.

### 2.2 Collection and preparation plant extract

The papaya plant (*Carica papaya*) leaves were collected from the premises of Noakhali Science and Technology University, Bangladesh. The fresh leaves were then washed multiple times with tap water followed by deionized water. The leaves were then dried in oven for an hour and then grinded to from fine powder. 20 grams of fine powders are boiled with 1L of deionized water at 80 °C for 30 min and the extract is then filtered using Whatman no 42 filter paper. The filtrate was concentrated using rotary evaporator and stored at 40°C for further use.

### 2.3 Green synthesis of α-Fe_2_O_3_ nanoparticles

Ferric chloride hexahydrate (FeCl_3_.6H_2_O) was used as the precursor for the synthesis of the α-Fe_2_O_3_ nanoparticles. 50 mL of the papaya leaves extract was added dropwise with 50 mL of 0.1M FeCl_3_.6H_2_O solution in 1:1 ratio at room temperature. Following this, 1 M NaOH was added till the pH became 11. The resultant mixture was stirred using a magnetic stirrer for 30 min and the formation of intense black colored solution confirmed the synthesis of iron oxide nanoparticles (Bibi et al., 2019). The nanoparticles were separated by centrifugation at 8000 rpm for 20 min and cleansed by subsequent washing with ethanol and water for 2–3 times. The NPs were finally dried in a hot air oven at 80 °C for 3 hr and stored in a seal tight container for further use.

### 2.4 Characterization

FT-IR spectra of sample was recorded on a FT-IR 8400S spectrophotometer (Shimadzu corporation, Japan) in the wavenumber range of 4000–400 cm^−1^. XRD patterns were recorded by an x-ray diffractometer (U1tima IV, Rigaku Corporation, Japan) by using Cu kα radiation (λ = 0.154) from a broad focus Cu tube operated at 40 KV and 40 MA. The morphology of nanoparticles was analyzed by means of field emission scanning electron microscope (JEOL JSM-7600F, Japan) run at a voltage of 5.0 KV. The UV-VIS spectra of the sample to measure the absorbance of the dye which were done by using the double beam UV-1700 Series Spectrophotometer (Shimadzu corporation, Japan).

### 2.5 Photocatalytic activity

To study the photocatalytic activity of nanoparticles, a series of remazol yellow RR dye solution having different concentrations (10 ppm, 30ppm, 50 ppm and 70ppm) were prepared. 100ml of the dye solution was taken in a beaker along with different quantity of nanoparticles (0.2 g/L, 0.4/g/L, 0.6 g/L and 0.8 g/L) and kept under sunlight along with continuous stirring. The absorbance was then taken after definite time intervals and the degradation efficiency was calculated by Eq. (1).

(1) $$Degradation\;\;efficicency\left(\%\right)=\frac{C_{0}-C_{t}}{C_{0}}X100$$

C_0_ is the initial concentration and C_t_ is the concentration at time t of remazol yellow RR dye.

The effect of pH on dye degradation was determined by maintaining a different pH environment (pH 2, 4, 6 and 8) using buffer solutions at optimum dye concertation and nanoparticles doses.

The time required for 25%, 50% and 75% degradation of dyes (T_25_, T_50_ and T_75_) were calculated from Eqs. (2), (3), and (4).

(2) $$T_{25}=\frac{0.288}{k}$$

(3) $$T_{50}=\frac{0.693}{k}$$

(4) $$T_{75}=\frac{1.386}{k}$$

Where, and k is the rate constant of the photocatalytic dye degradation reaction.

### 2.6 Antibacterial activity

Well diffusion method by agar plates was used for calculating the zone of inhibition (Shamaila et al., 2016). Clinical pathogenic bacteria *Klebsiella* spp*.*strain KH15*, E.Coli* strain EH9*, Pseudomonas* spp. strain PsI1strain PsI1*, S.aureus* strain 6s were developed on nutrient agar plate and maintained at 37 °C for whole night. The overnight culture of bacteria in nutrient broth (Oxoid Limited, United Kingdom) was used for the experiment. In this method, sterilized nutrient agar plate was equipped for each bacterium. All bacterial culture was adjusted in optical density (OD) 0.1 using UV–Visible Spectrophotometer (Spectrumlab 1200RS, Japan). The spectrophotometer was first made auto zero using blank to eliminate the effect of assay reagents. These three bacterial pathogens were then coated over the agar plate with the help of sterile swab of cotton. Then these plates were permitted to dry. After that, one wells were bored by a sterile well cutter (7.0 mm diameter) in each agar plate. Subsequently, the suspension of NPs (5 mg/ml, 20 mg/ml and 30 mg/ml) was poured into individual wells for each strain. The plates were permitted to put for 1 h for complete diffusion followed by incubation at 37 °C for 24 h (hr) and measured the diameter of inhibitory zones in mm.

### 2.7 Cytotoxicity study

Cytotoxic effect of α-Fe_2_O_3_ nanoparticle was observed against Hela, BHK-21 and Vero cell line that were collected from Centre for Advanced Research in Sciences (CARS) of University of Dhaka. Hela, a human cervical carcinoma cell line, BHK-21, a baby hamster kidney fibroblast cell line and Vero cell line, a kidney epithelial cells of African green monkey, were maintained in DMEM (Dulbecco's Modified Eagles' medium) containing 1% penicillin-streptomycin (1:1) and 0.2% gentamycin and 10% fetal bovine Serum (FBS). Hela cells (2×10^4^/100 μl) and BHK-21 cells (1.5×10^4^/100 μl) were seeded onto 96-well plate and incubated at 37 °C in CO_2_ incubator (Nuaire, USA). 25 μl (30 mg/mL) sample of nanoparticles (autoclaved) was added each well. After 48 h of incubation, insoluble samples were washed out with fresh media and cytotoxicity was examined under a Trinocular microscope with camera (Optika, Italy). Duplicate wells were used for each sample.

### 2.8 Ethical statement

This research work did not include any animal. We carried out antibacterial activity on different bacterial reference strains that we mentioned. We also carried out cytotoxic activity using 3 different kind of cell lines that are commercially available. There was no necessity for ethical approval consideration in this study.

## 3 Results and discussions

### 3.1 Preparation of α-Fe_2_O_3_ nanoparticles

**Figure 1:**
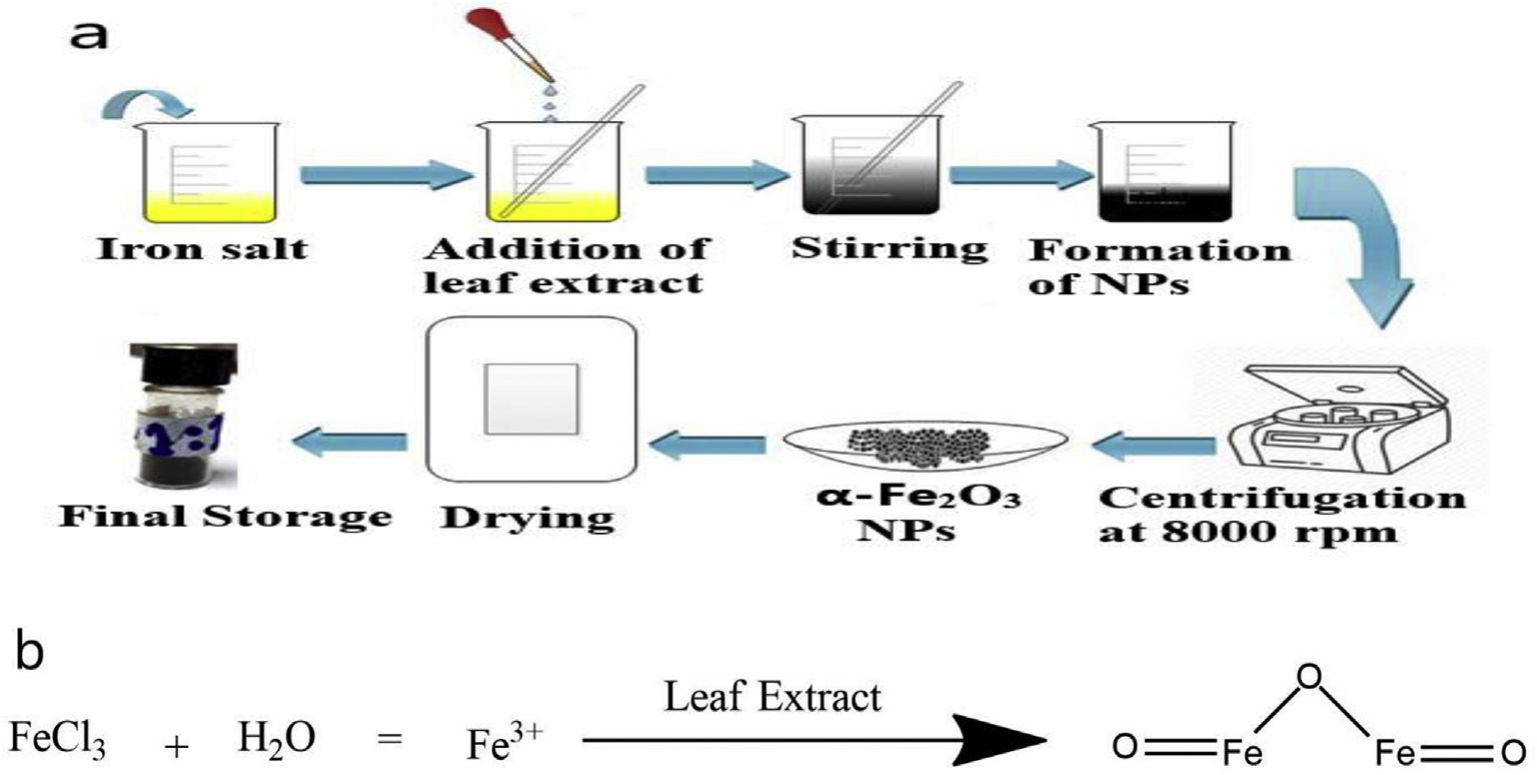
(a) Synthesis route of α-Fe_2_O_3_ nanoparticles and (b) possible reaction mechanism for synthesis of α-Fe_2_O_3_ nanoparticles.

The synthesis scheme of iron oxide NPs is shown in Figure 1a. The various phytochemicals like polyphenols, flavonoids, glycosides, and tannins present in the leaf extract equally act as reducing and stabilizing agents for the synthesis of NPs (Juárez-Rojop et al., 2014). The formation of black color precipitates occurred due to the interaction between these phytochemicals and metal ions ensuring the formation of α-Fe_2_O_3_ nanoparticles (Anchan et al., 2019). After mixing of iron salt with leaf extract at definite reaction condition, it is not be able to reduce Fe^3+^ to Fe^0^; rather, the phytochemicals react with the iron ions to give iron oxide NPs, as it is prone to oxidation (Figure 1b) (Devi et al., 2019).

### 3.2 Characterization

**Figure 2:**
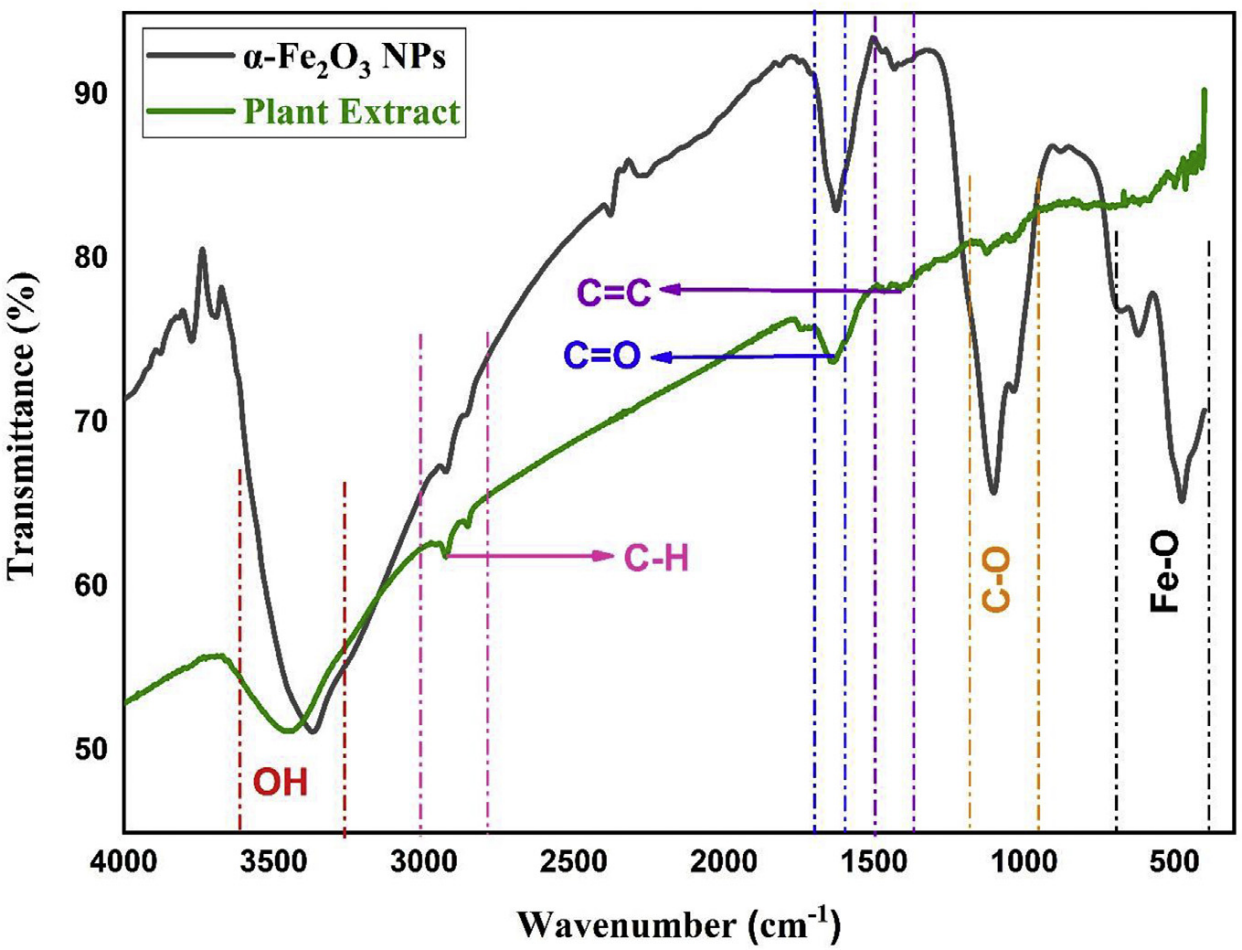
Vibrational properties of plant extract mediated synthesized iron oxide nanoparticles.

The FTIR analysis (400-4000 cm^−1^) of the synthesized sample ensured the synthesis of α-Fe_2_O_3_ nanoparticles as well the existence of various reducing agents functional groups presents in the papaya plant leaf extract (Figure 2). The peaks at 474.49 cm^−1^, 621.08 cm^−1^ and 678.94 cm^−1^ ensure the presence of Fe–O bond in the sample (Aisida et al., 2020; Liu et al., 2009). The peaks at position of 3357.57 cm^−1^ represent the –OH bond stretching from aqueous phase. Again, the peaks at 1433.11 cm^−1^, and 3691.57 cm^−1^ also represents the –OH bond stretching and bending from various phenolic and carboxylic group respectively present the plant extract (Qasim et al., 2020). Moreover, the peaks at 2927.94 cm^−1^, 1625.99 cm^−1^ and 1101.35 cm^−1^ denotes C–H stretching, C=C stretching and C–O stretching ensuring the presence of alkane, conjugated alkene and secondary alcohol in the plant extract correspondingly as observed by other study (Aisida et al., 2020). The shift in peak position in the range of 400–4000 cm^−1^ ensure that these functional groups containing compounds bound to the iron oxide surface.

**Figure 3:**
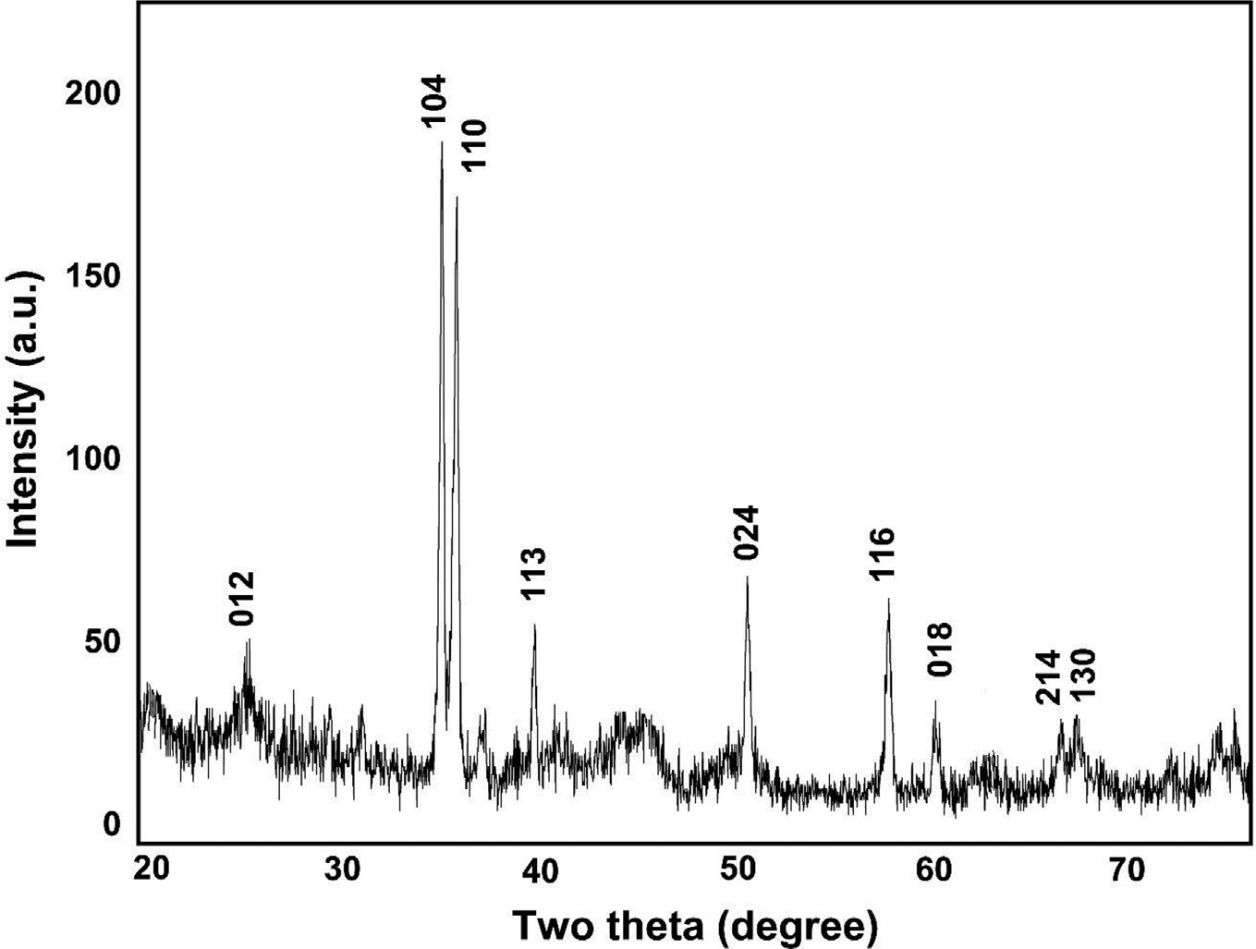
XRD data of α-Fe_2_O_3_ nanoparticles.

The XRD data as shown in Figure 3 indicates that the crystal planes of (012), (104), (110), (113), (024), (116) and (018) corresponding to the of 25.16°, 35.12°, 36.63°, 40.64°, 49.97°, 57.08°, and 59.42° indicates the formation of α-Fe_2_O_3_ nanoparticles. The intense and sharp peaks undoubtedly revealed that Fe_2_O_3_ nanoparticles formed by the reduction method using *Carica papaya* leaf extract were crystalline in nature. The results are almost similar to the results obtained for iron oxide nanoparticles by other researchers (Ahmmad et al., 2013; Suresh et al., 2016; Lassoued et al., 2018). The average crystallite size as determined using the Debye–Scherrer equation was found to be 4.58 nm.

**Figure 4:**
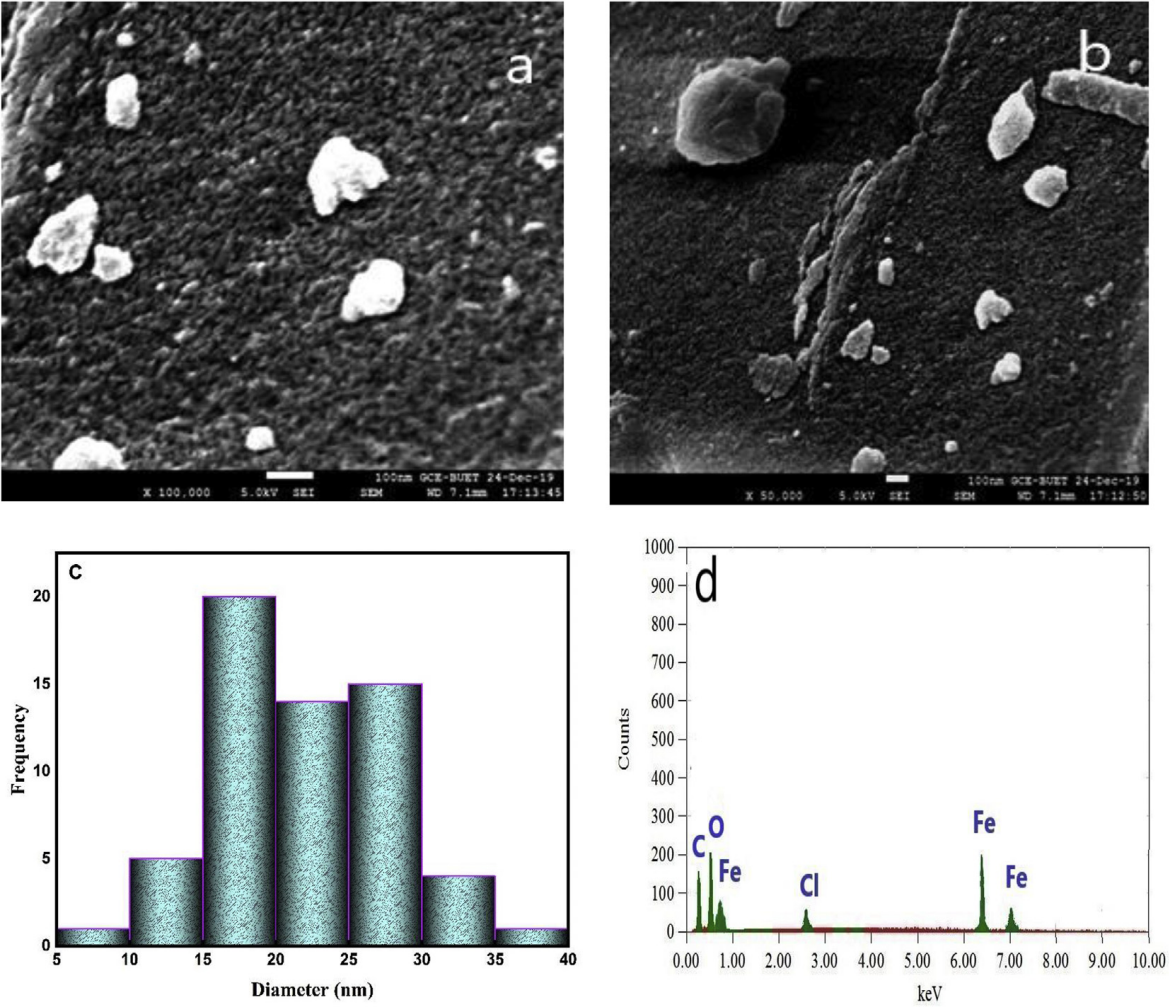
(a–b) FESEM image showing morphology; (c) Size distribution histogram for α-Fe_2_O_3_ nanoparticle (d) EDX data of green synthesized α-Fe_2_O_3_ nanoparticles.

The morphology of the synthesized nanoparticle is shown in Figure 4 (a-b). The figure indicates that the synthesized nanoparticles are not uniform in nature and get agglomerated in some cases. The size of NPs was determined by selecting 100 particles and their average diameter was found to be 21.59 nm (Figure 4c). The big agglomerated clusters were formed due to accumulation of tiny building blocks of various bioactive reducing agents of plant extract or this might be due to the lower capping ability of the plant extract and agglomeration tendency of the iron-based nanoparticles due to magnetic interactions.

Furthermore, the elemental composition of the sample was analyzed by EDX analysis. The EDX analysis reported in Figure 4c, clearly shows the presence of the K-α at 6.4 keV due to Fe atoms present in the nanoparticle and two K-α lines at 0.28 keV and 0.6 keV coming from the C and O atoms respectively. Similar results were also obtained by other study (Vasantharaj et al., 2019). The percentage of mass present under the irradiated area is 25.05%, 31.03%, 32.70% for Carbon, Oxygen, and iron respectively (Table 1). The presence of carbon is from the plant extract components existing in the NPs surface (also shown in FTIR data) and the presence of Cl as impurities is usually observed during iron oxide NPs synthesis from ferric chloride as observed by others (Qasim et al., 2020). Furthermore, the negative zeta potential data (-15.1 mV) revealed that the synthesized NPs is stable and its stability remains almost constant over thirty days after its synthesis (Table 1).

**Table 1:** EDX and zeta potential data of α-Fe_2_O_3_ nanoparticles.

EDX data		Zeta potential (ζ)	
Element	Mass%	Day	mV
C	25.05	0	-15.1
O	31.03	15	-14.8
Fe	32.70	30	-14.4

**Figure 5:**
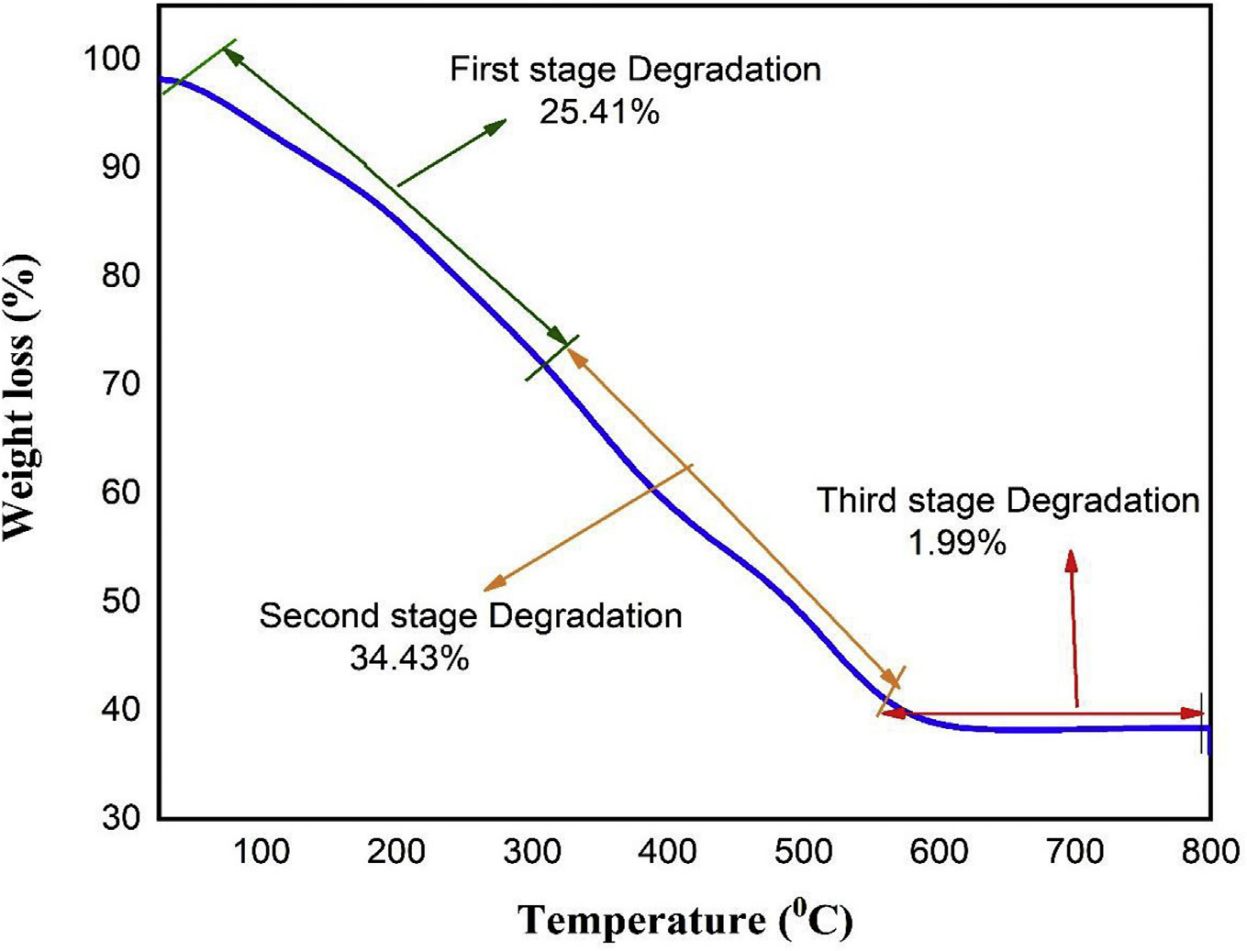
TGA data of green synthesized α-Fe_2_O_3_ nanoparticles.

TGA analysis was done to know the thermal stability, decomposition temperature, and also decomposition rate of the nanoparticles. TGA measurements were performed with a heating rate of 10 °C/min in the temperature range of 24.38 °C to 800 °C. The TGA curve of the iron oxide nanoparticle is shown in Figure 5. The TGA plot depicted three weight loss steps in the tested temperature range of 24.38 °C to 798.70 °C. The first step 25.41% weight loss occurred in the temperature range of 24.38 °C to 301.70 °C indicating the removal of water, organic solvent or residual solvent, physiosorbed and chemisorbed H_2_O molecules in the sample. In second and third steps of TGA curve, total of 34.43% weight loss occurred at 301.70 °C to 798.70 °C suggesting the elimination or decomposition of the capping biomolecules. Further, there was no weight loss observed above 798.70 °C and 40.16% weight residue of the iron oxide nanoparticles remained at 798.70 °C.

### 3.3 Degradation study of remazol yellow RR dye

To study the efficiency of synthesized nanoparticles as a photocatalyst, we used remazol yellow RR as a model dye. The experiment was conducted under sunlight irradiation during 8 am to 4 pm every day. To complete the degradation process time, we kept the sample of current day in dark place to keep it again under sunlight in the next day if necessary. For that reason, we also experimented the degradation under dark place and we did not observe any degradation. However, to optimize the dye degradation we maintained the degradation process with different doses of photocatalyst, time, initial dye concentration and pH value.

#### 3.3.1 Influence of contact time and catalyst loading

**Figure 6:**
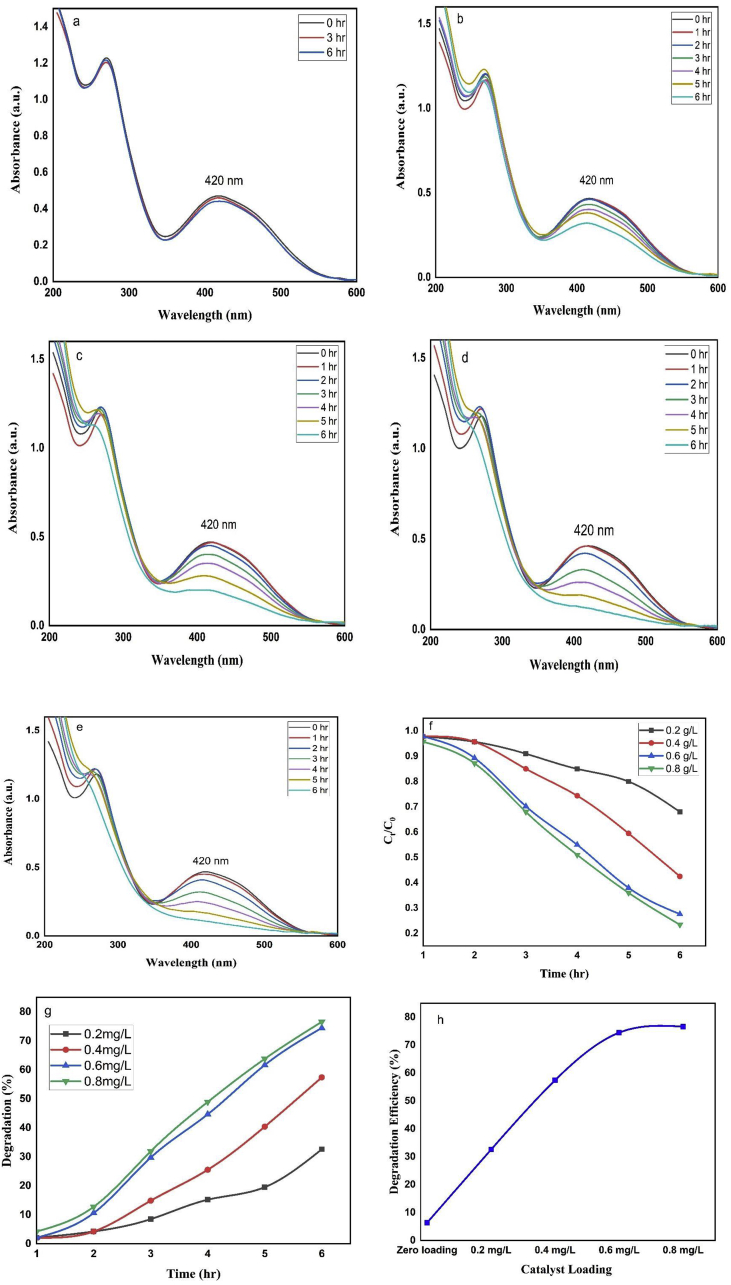
Degradation of remazol yellow RR dye (a) in absence of photocatalyst; in presence of (b) 0.2 g/L, (c) 0.4 g/L, (d) 0.6 g/L, (e) 0.8 g/L; (f) extent of degradation with time at different catalyst doses with time; (g) degradation efficiency at different time intervals (h) influence of catalyst loading on degradation of dye.

To study the effect of contact time and catalyst loading, a series of experiments were carried out for 6 hr by varying the nanoparticles doses from 0.2 to 0.8 g/L keeping the dye concentration at 50 ppm and pH of 2 as shown in Figure 6(b-e). To evaluate the nanoparticle activity a blank experiment was also done represented in Figure 6a. From Figure 6(a-f), it is very clear that without using nanoparticles there was no significant degradation over a period of 6 hr, whereas with the increasing amount of catalyst loading the there was a sharp increase in degradation rate with reaction time. As shown in the figure, for all catalyst doses the degradation increased with time and for 0.4 g/L of catalyst dose, the degradation reached to about 2, 4, 14, 25, 40, 57% after1 2, 3, 4, 5, and 6 h respectively (Figure 6g). Again, the results in Figure 6h, demonstrate the increase in catalyst dose is directly proportional to the photocatalytic dye degradation percentage. More specifically, without photocatalyst the degradation was only 6.38%, for 0.6 g/L doses the degradation was 74% and for maximum catalyst dose (0.8 g/L) the degradation almost reached to 77% after 6 hr as shown in Figure 6h. The lower degradation rate at lesser catalyst dose was due to the less amount of photon absorption by the catalyst and the subsequent decrease in ROS production. While with a continuous increase in catalyst (for 0.6 and 0.8 g/L), the degradation efficiency became almost constant (Figure 6g-h) which might be due to the opacity of solution, agglomeration of nanoparticles and light scattering ability of nanoparticles at higher catalyst dose (Reza et al., 2017). Therefore, it can be concluded that neither a higher nor a lower catalyst dose is suitable for the degradation of reactive dyes, therefore we selected a dose of 0.4 g/L for further studies.

#### 3.3.2 Degradation kinetics

The kinetic constants of dye photodegradation are usually estimated by applying a pseudo-first-order and second-order reaction rate Eqs. (5) and (6).

(5) $$\text{ln}\left(\frac{{\text{C}}_{\text{o}}}{{\text{C}}_{\text{t}}}\right)={\text{K}}_{1}\text{t}$$

(6) $$\frac{1}{{\text{C}}_{\text{t}}}-\frac{1}{{\text{C}}_{0}}={\text{ K}}_{2}\text{t }$$

Where t (hr) is the reaction time, C_0_ is the initial concentration, C_t_ is the concentration at time t, k_1_ (hr^−1^) and k_2_ (Lmg^−1^hr^−1^) represents the first-order and second-order reaction rate constant.

**Figure 7:**
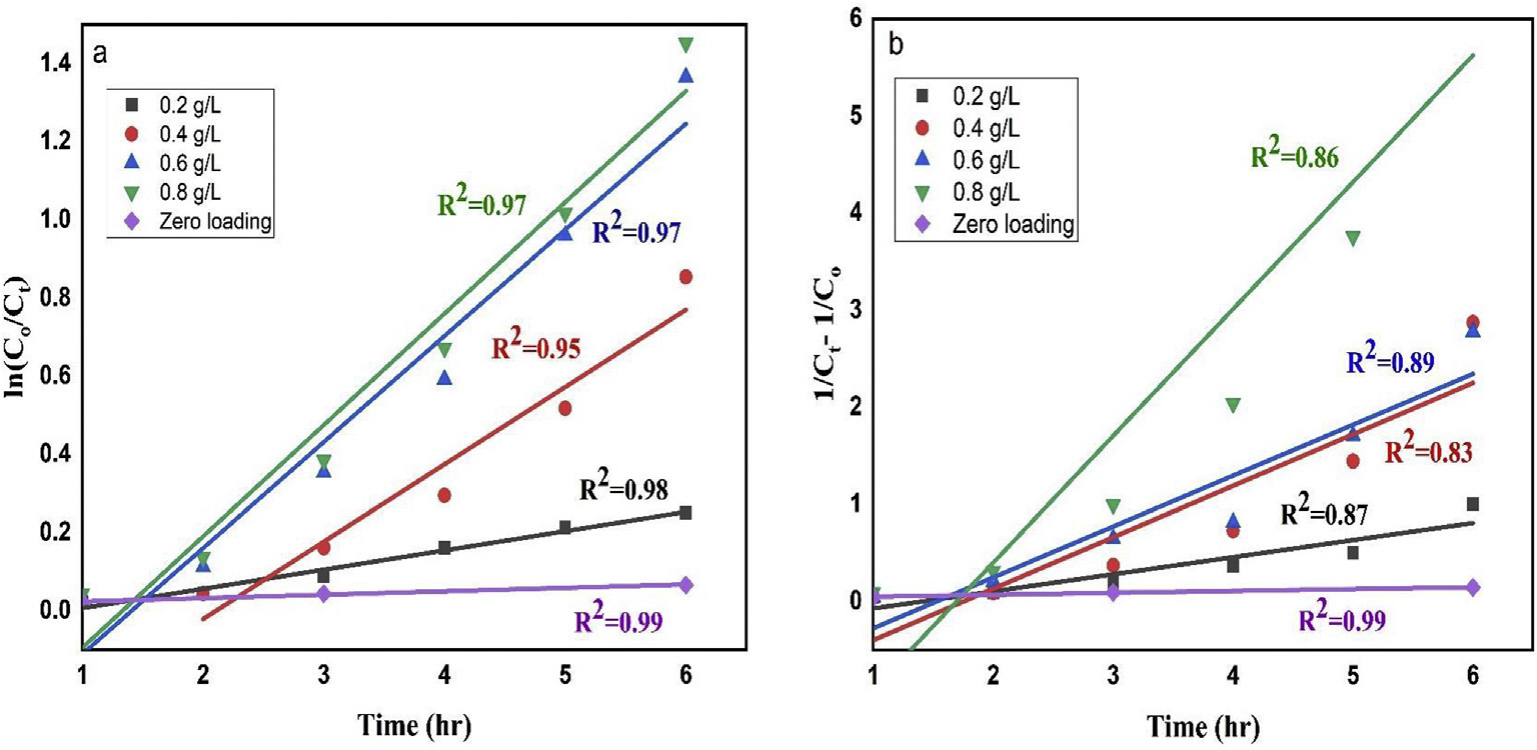
The (a)first-order and (b) second-order linear plot of ln (C_0_/C_t_) vs. time for remazol yellow RR dye degradation in the presence of nanoparticles as catalyst.

The rate constant (k) of first-order reaction was obtained by plotting ln (C_0_/C_t_) against the reaction time t and 1/C_t_- 1/C_o_ against time (t) for second-order reaction as shown in Figure 7(a-b). Figure showed that the degradation of dye is completely dose dependent and fits perfectly with first-order kinetics (R^2^ value of first-order reaction is higher than second-order reaction for all doses). The lowest degradation rate of 0.008 ± 0.0009 hr^-1^ (R^2^ = 0.99) was observed for blank sample (without NPs) while the rate constant increased from 0.06 to 0.33 hr ^−1^ for 0.2–0.8 g/L of catalyst doses which indicates that the reaction rate is almost forty times higher in case of 0.8 g/L of catalyst as compared to the absence of NPs (Table 2). However, the dye degradation reached its half value by 3.47 h for 0.4 g/L doses while it took only 3.47 hr and 2.57 hr for 0.6 g/L and 0.8 g/L of catalyst doses correspondingly. Furthermore, although 0.2 g/L of catalyst could not cause 75% of dye degradation within 6 h period but it was completed only in 5.13 and 4.27 hr by 0.6 g/L and 0.8 g/L of catalyst doses respectively.

**Table 2:** Comparison of degradation kinetics of dye solution by nanoparticles for 6 hr.

Nanoparticle Catalyst	Parameters	Remazol yellow RR dye
Zero loading	First-order rate constant (k) (hr^−1^)	0.008 ± 0.0009
	T_25_ (hr)	-
	T_50_ (hr)	-
	T_75_ (hr)	-
0.2 g/L NPs	First-order rate constant (k) (hr^−1^)	0.06 ± 0.004
	T_25_ (hr)	4.79
	T_50_ (hr)	-
	T_75_ (hr)	-
0.4 g/L NPs	First-order rate constant (k) (hr^−1^)	0.20 ± 0.03
	T_25_ (hr)	1.44
	T_50_ (hr)	3.47
	T_75_ (hr)	-
0.6 g/L NPs	First-order rate constant (k) (hr^−1^)	0.27 ± 0.03
	T_25_ (hr)	1.07
	T_50_ (hr)	2.57
	T_75_ (hr)	5.13
0.8 g/L NPs	First-order rate constant (k) (hr^−1^)	0.33 ± 0.02
	T_25_ (hr)	0.87
	T_50_ (hr)	2.10
	T_75_ (hr)	4.20

**Figure 8:**
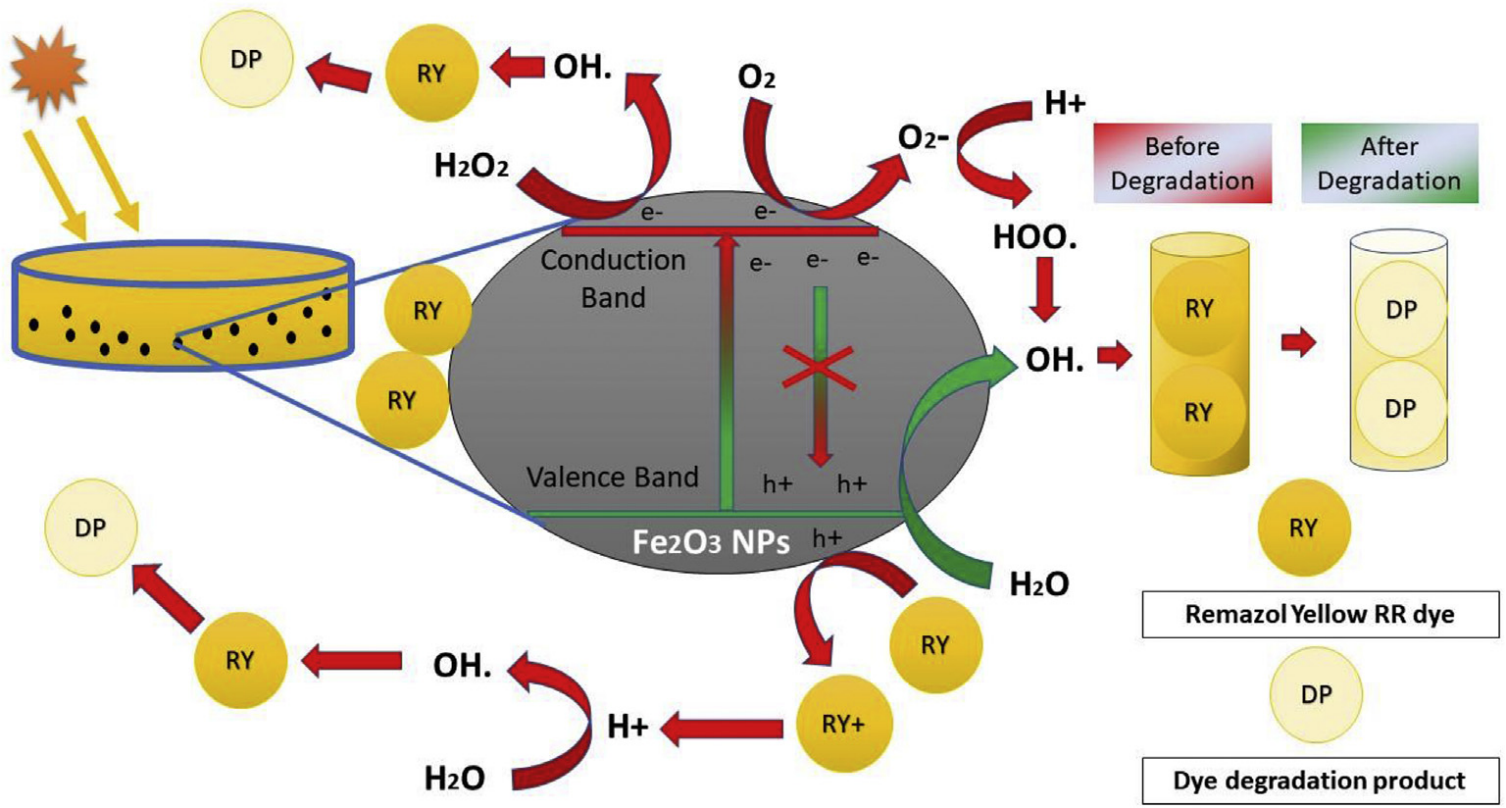
Probable degradation mechanism of remazol yellow RR dye by iron oxide NPs under sunlight irradiation.

Iron oxide NPs produce hydroxyl radical (•OH) under sunlight radiation which are reposnsible for the degradation of remazol yellow RR dye and the probable degradation mechanism is shown in Figure 8. Generally, when NPs were irradiated under sulight, an electron (e^−^) and hole (h^+^) pair is produced (Varadavenkatesan et al., 2019, Varadavenkatesan et al., 2019). The produced electron is excited from the valence band to the conduction band, leaving the h^+^ in the valence band. This hole (h^+^) is responsible for the conversion of water into hydroxyl radical, which is responsible for oxidative degradation of dye. On the other hand, electron combines with molecular oxygen and converted into superoxide radical. The superoxide radical is further converted in to hydroxyl radical which is a strong oxidizing agent and degrades the dye to harmless end products (Kamaraj et al., 2019). Again, the highly oxidizing hole generated by NPs after absorbing the sunlight causes the direct oxidation of dyes and release H+ ions which further get united with water to give oxygen reactive species and OH^־^ that help in degradation of the dye (Kansal et al., 2009). Furthermore, the various biomolecules present in plant extract and even in the NPs surface acts like catalysts to boost the photocatalytic activity and the subsequent enriched degradation of dye molecules (Haritha et al., 2016).

#### 3.3.3 pH effect

**Figure 9:**
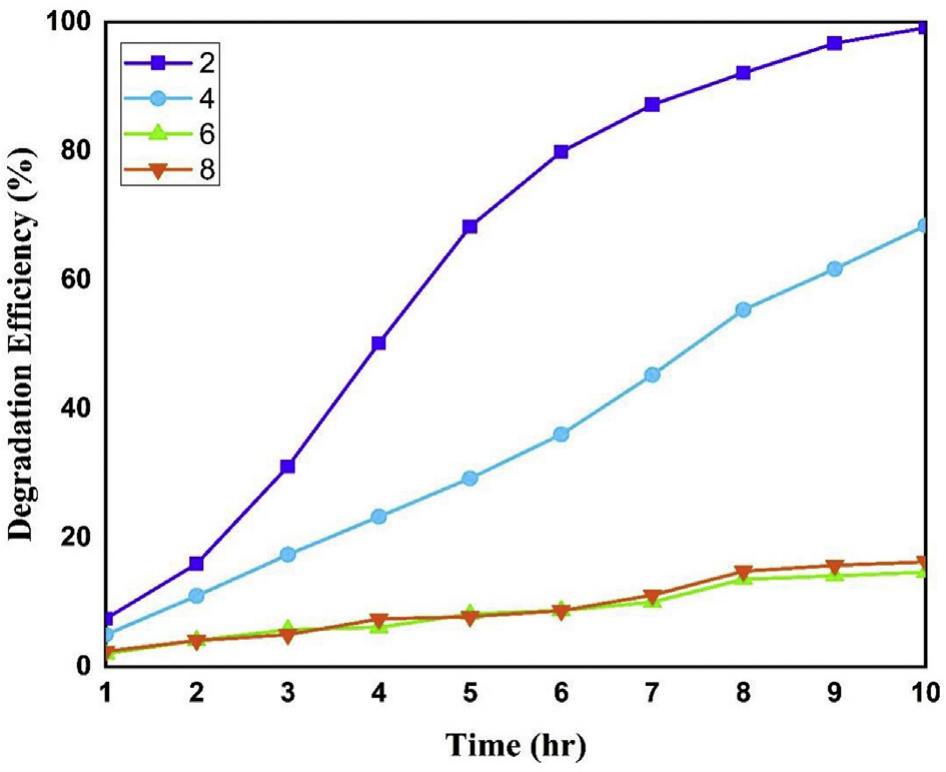
Effect of pH on photodegradation of remazol yellow RR dye.

To study the effect of pH effect over dye degradation a number of experiments were done within a pH range 2–8 maintaining 0.4 g/L of catalyst doses and 50 ppm of dye concentration (Figure 9). The pH of the solution was adjusted before sunlight irradiation and is not controlled during the degradation reaction. From Figure 9 it is clear that the degradation was favored at lower pH while it was found to be decreased with the increase in pH value. More specifically, the highest degradation of almost 99% was observed at pH 2, followed by 68% degradation at pH 4 while for the rest of the pH region the degradation almost remained same with the highest value of about 15% within 10 hr. The degradation of dye usually depends on the adsorption of dye molecules on the catalyst surface and formation of hydroxyl free-radical which in turn depends on the availability of hydroxyl ion in the reaction medium. The zero-point charge (pH_zpc_) of iron oxide is between 6-7 which means that below this range it becomes positively charged and above this range it becomes negatively charged (Meng et al., 2016). So, at lower pH value (particularly at 2) attractive forces between the NPs surface (positively charged) and the anionic remazol yellow RR dye favors adsorption resulting in the enhanced degradation. The lower degradation rate at neutral pH was observed because, close to zero-point charge (pH_zpc_) region the iron oxide nanoparticles get agglomerated and resulting in the decreased amount hydroxyl radical formation (OH·) and a subsequent decrease in degradation rate. Furthermore, although higher pH value could provide a higher concentration of hydroxyl ions to react with valence band holes (h^+^) to form more OH·, but the negative surface charge of NPs resulting in electrostatic repulsion, which keep away the hydroxyl ion and oxygen molecule from adsorbing on its surface and thus decreases the availability of hydroxyl and superoxide radical for dye degradation (Choudhury et al., 2012). Again, at higher pH deterioration of H_2_O_2_ into oxygen and water rather than hydroxyl radical (OH·) become favorable resulting in a decrease in dye degradation (Shu et al., 2004). However, it is shown form the figure that, although more than 75% of the dye (at pH 2) was degraded within 6 hr but it took four more hours for its complete degradation, which means that the degradation become slow after 6 hr. This has happened because the surface of NPs might get occupied with the dye molecules which restrains the NPs from further degradation of dye molecules.

#### 3.3.4 Initial dye dose variation

**Figure 10:**
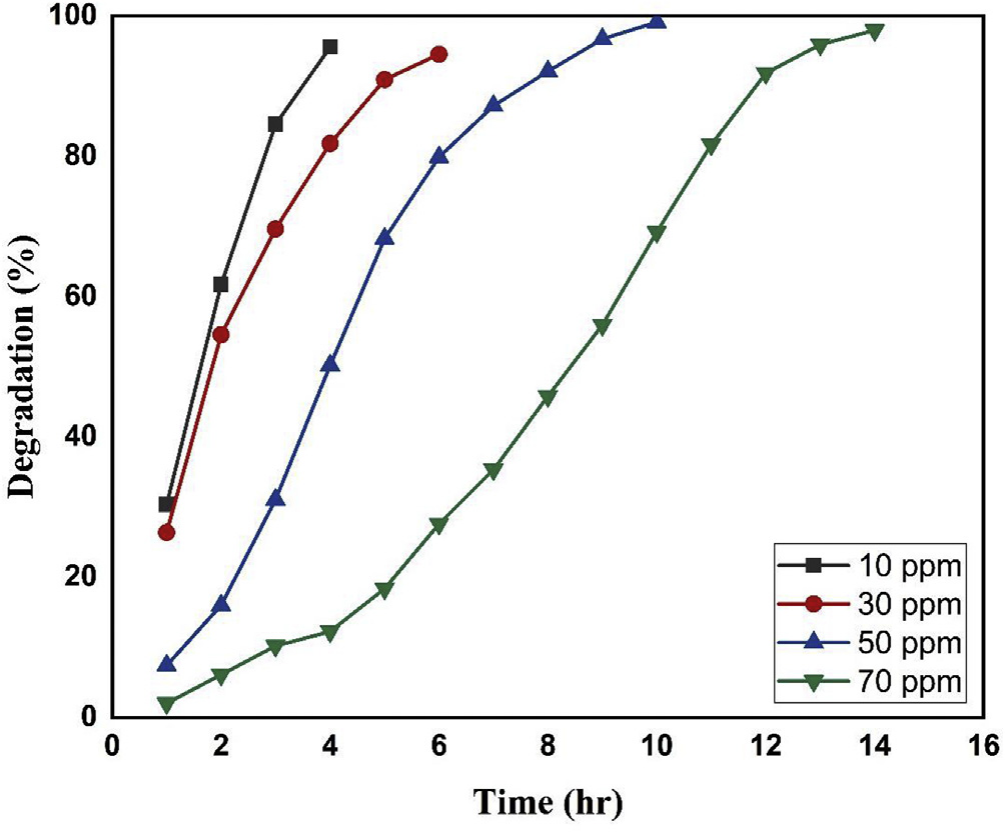
Influence of initial dye concentration on the degradation efficiency of dye by NPs.

The effect of initial dye dose variation on the photocatalytic degradation efficiency of dye by iron oxide NPs was examined by changing the dye concentration from 10 to 70 mg/L at constant catalyst loading of 0.4 g/L and pH 2. As shown in Figure 10, it is very clear that the degradation efficiency decreases with the increase in initial due concentration. As indicated in figure, the 10ppm solution reached its maximum degradation within 4 h, 30 ppm dye reached its maximum degradation within 6 hr and it took about 14 hr for almost complete degradation of 70 ppm dye solution for the same amount of catalyst doses. This is because, at higher dye concentration, dye molecules might adsorb on the catalyst surface, hence a significant amount of UV light is absorbed by the dye molecules themselves rather than by the nanoparticles which in turns results in the decrease in the generation of hydroxyl radicals and dye degradation (Daneshvar et al., 2003). Also, the intermediates formed during photocatalytic degradation process might compete with the intact dye molecules for the available active sites on the catalyst surface (So et al., 2002). Actually, with the increase in initial dye concentration the necessity of catalyst surface for degradation also increases. Since both the sunlight irradiation time and catalyst dose are constant, the hydroxyl radical (•OH) formed on the surface of the catalyst is also constant and insufficient for the degradation of excess dye.

#### 3.3.5 Unit removal capacity (URC)

**Figure 11:**
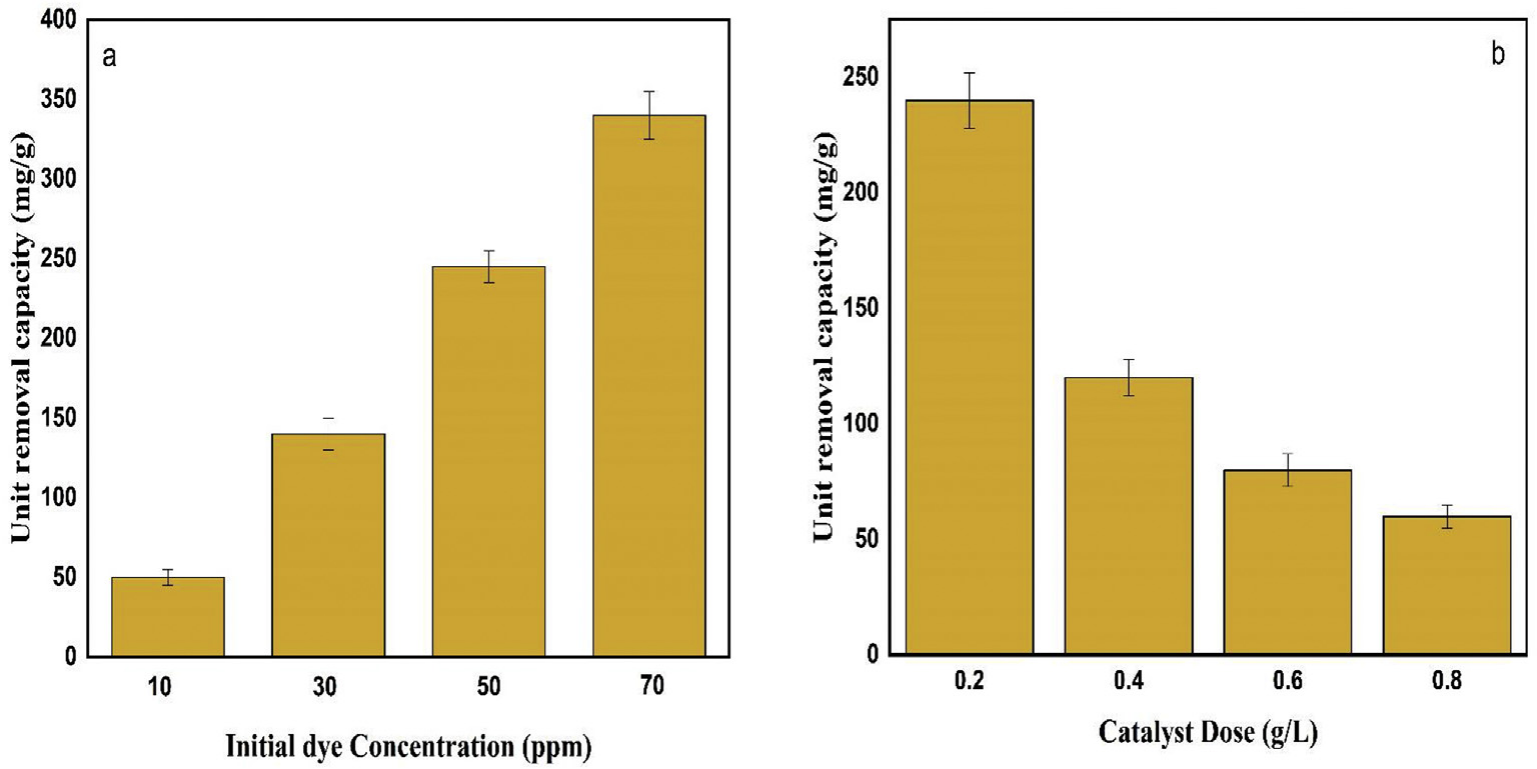
Unit removal capacity of photocatalyst with respect to (a) initial dye concentration and (b) catalyst doses. Date are presented as mean ± SD.

The unit removal capacity of photocatalyst was calculated to determine the removal capacity of catalyst per unit of weight. From Figure 11 (a-b) it is shown that the URC value was increased sharply with the increase in initial dye concentration and it showed an inverse trend in case of catalyst dose variation for the same initial dye concentration. Each gram of nanoparticles removed 50, 140, 245 and 340 mg of dye in case of 10, 30, 50 and 70 ppm of initial dye concentration respectively. On the other hand, for the same initial dye concentration (50 ppm) each gram of photocatalyst remove 240, 120, 80 and 60 mg for 0.2, 0.4, 0.6 and 0.8 g/L of catalyst doses correspondingly. The increase in URC with the increase in initial dye concentration was due to the availability of dye for degradation while the decrease in URC with increasing catalyst dose might be due to the unsaturation of active sites of catalyst.

#### 3.3.6 Comparison with other works

A comparison on degradation of various reactive dyes by several NPs is summarized in Table 3. Iron oxide NPs synthesized by green reduction method showed activity against safranin dye with degradation (68.8%) within 180 min (Qasim et al., 2020). In case of degradation of remazol yellow RR dye by TiO_2_ NPs in presence of H_2_O_2_ it took about 15 min for 98% degradation with a catalyst doses of 1 g/L (Bibi et al., 2017). The NPs synthesis in this study showed excellent activity without any additives and with a lower catalyst dose as well as compared to other studies (Kumar et al., 2018).

**Table 3:** Comparison of reactive dyes degradation efficiency by various NPs.

Nanoparticles	Synthesis route	Catalyst Dose (g/L)	Dye	Dye dose (ppm)	Degradation (%)	Time (min)	Reference
FeO nanorods	Green reduction	0.005	Safranin	10	68.8	180	(Qasim et al., 2020)
FeO nanorods	Chemical Reduction	0.005	Safranin	10	24.82	180	(Qasim et al., 2020)
FeO NPs	Green reduction	-	Crystal violet	10	78.78	150	(Vasantharaj et al., 2019)
α-Fe_2_O_3_ NPs	Green reduction	0.8	Remazol yellow RR	50	75	250	This study
TiO_2_ NPs with H_2_O_2_	Chemical method	1	Remazol yellow RR	75	98	15	(Soutsas et al., 2010)
Cobalt Oxide NPs	Green reduction	0.005	Remazol brilliant orange 3R	150	78.45	50	(Bibi et al., 2017)
Tin Oxide NPs	Green reduction	1	Reactive yellow 186	40	90	180	(Kumar et al., 2018)
Silver NPs	Green reduction	2.5	Methylene blue in presence of NaBH_4_	40	80	20	(Paul et al., 2020)

### 3.4 Antimicrobial activity of α-Fe_2_O_3_ NPs

**Figure 12:**
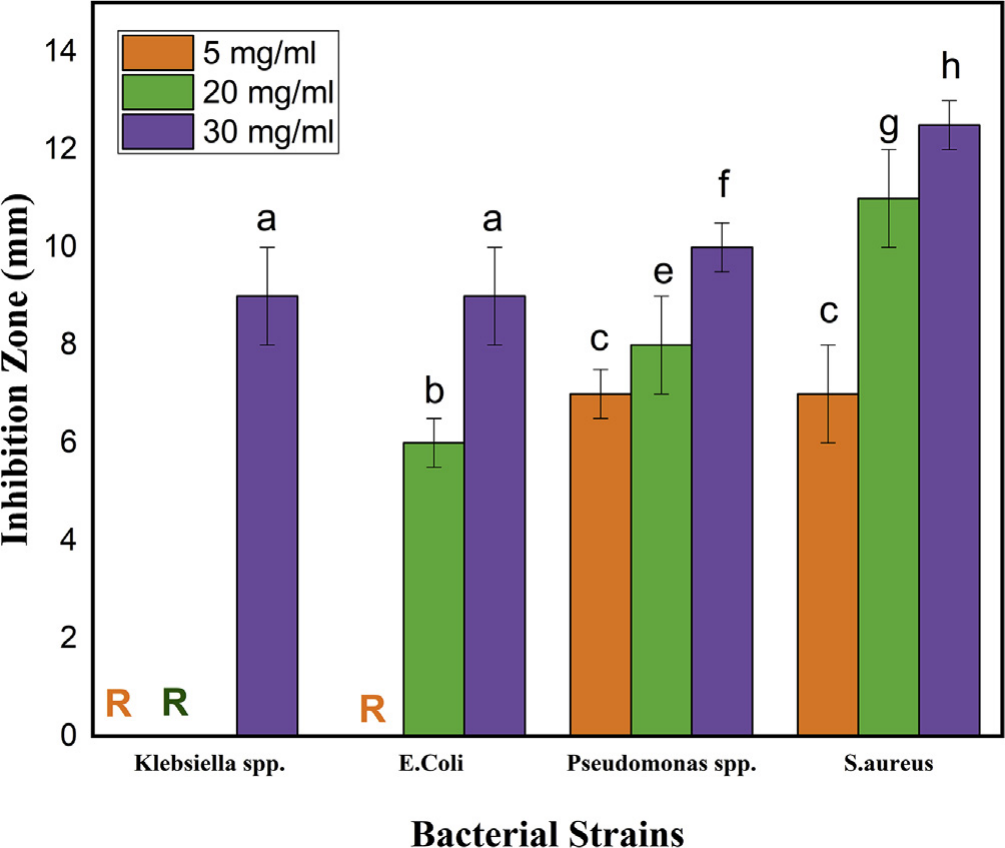
Antibacterial inhibition zone of α-Fe_2_O_3_ nanoparticles at different concentration. Date are presented as mean ± SD and analyzed with one-way analysis of variance. Different letters represent statistical significance among different treatments (p < 0.05). The symbol “R” indicates that those are resistant to studied bacterial strains.

The antibacterial activity of green synthesized nanoparticles suspensions of different concentrations was done against three gram negative bacteria such as *Klebsiella* spp.*, E.Coli, Pseudomonas* spp.*,* and one gram positive bacteria which is *S.aureus* in aqueous lysogeny broth (LB). The well diffusion method was used to test the ability of the antibacterial agent (NPs) to rupture the bacterial cells. The antibacterial activity studied against gram-positive and gram-negative bacteria at different concentrations of samples are shown in Figure 12.

From Figure 12 it is very clear that the synthesis nanoparticles showed antibacterial properties and the highest effect was observed for *S. aureus* while the lowest effect was for the *Klebsiella* spp. whereas the activities are completely dose dependent. For *S. aureus* the zone of inhibition was 7 ± 1 mm for 5 mg/ml doses while it gets almost doubled to an inhibition zone of 12.5 ± 0.5 mm for 30 mg/ml. On the other hand, *Klebsiella* spp. showed resistance to 5 mg/ml and 20 mg/ml doses but at 30 mg/ml doses it grown an inhibition zone of about 9 ± 1 mm. Moreover, the NPs exhibited moderate effect on both *E.Coli* and *Pseudomonas* spp. with an inhibition zone of about 9 ± 1 mm and 10 ± 0.5 mm respectively at 30 mg/ml dose. The as possessed antibacterial properties of nanoparticles is due to its nanoscale size allowing to accumulate or deposit on the surface of studied bacterial strains which is reported by other researchers (Varadavenkatesan et al., 2019, Varadavenkatesan et al., 2019; Jagathesan and Rajiv, 2018; Groiss et al., 2017).

Apart from the NPs, the plant extracts might also possess antibacterial activity due to the presence of phytochemical components (Vasantharaj et al., 2019). However, there are a number of hypotheses involved to explain the exact mechanism of NPs against bacterial strains. Generally, the iron oxide nanoparticles showed its antibacterial properties due to the production of reactive oxygen species (ROS), oxidative stress caused by ROS, the reaction of ions released by nanoparticles with thiol groups (–SH) of the bacterial cell, therefore, interrupt the DNA replication and protein synthesis process of microorganism by altering their structure (Arakha et al., 2015; Luo et al., 2015). Again, the positively charged metal ions released from NPs might interact with negatively charged bacterial strains surface with a result in the disruption and destabilization of microorganism surface protein and subsequent cell death (Cardillo et al., 2016). However, this study also suggested that the nanoparticles showed better activity against gram positive bacteria as compared to the gram-negative strains. This has happened because the gram-negative bacteria usually own an extra outer layer of lipopolysaccharide and peptidoglycan which helps the gram-negative bacteria to reduce the damage that might cause by nanoparticles.

### 3.5 Cytotoxicity study

**Figure 13:**
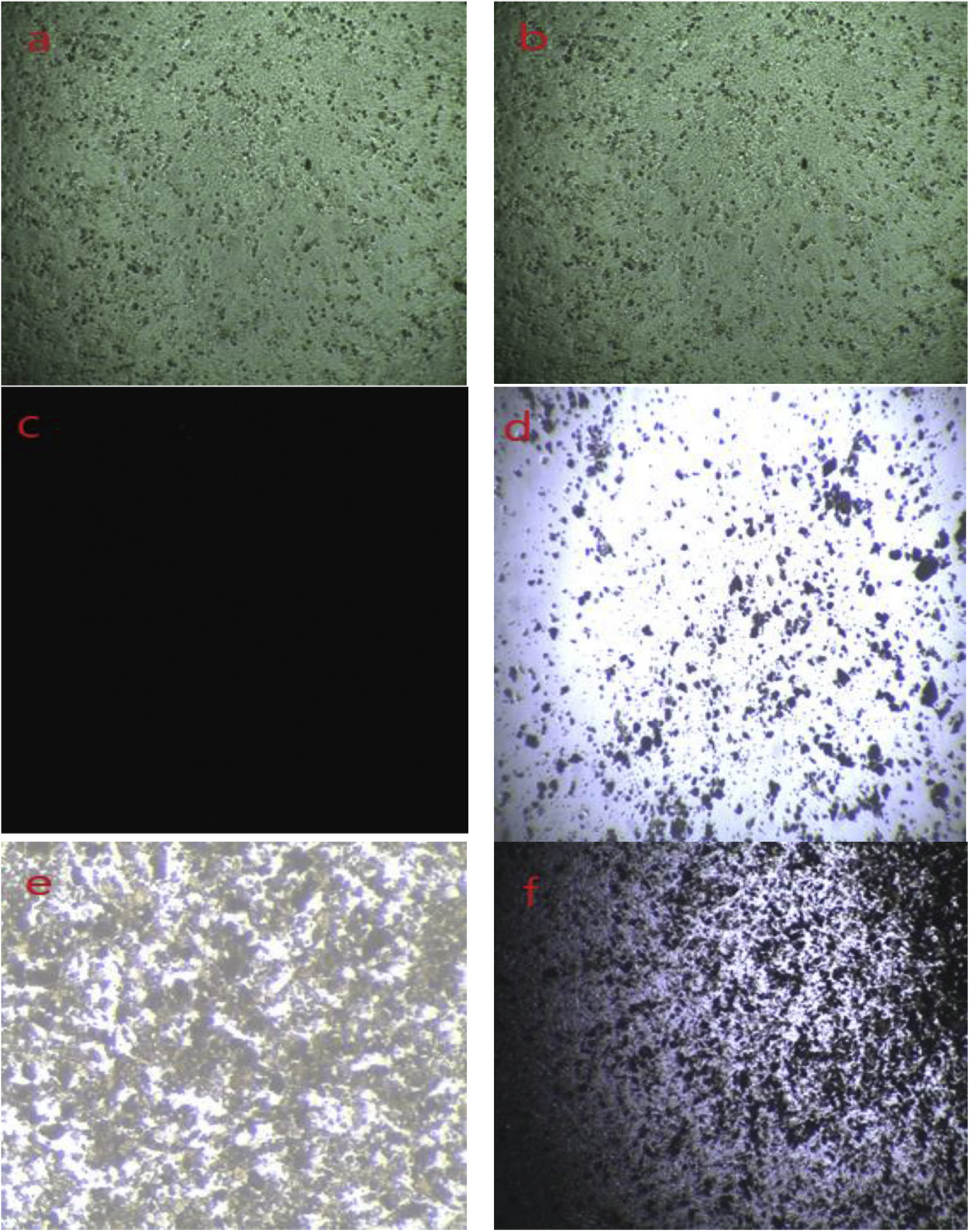
(a) and (b) represents the cell line effect in absence and presence of solvent in the medium respectively; (c) represents the cell line before washing with fresh media. *In vitro* cytotoxicity of NPs on (d) Hela cell line; (e) BHK-21 cell line; (f) Vero cell line.

Cytotoxic effect was examined against Hela, a human cervical carcinoma cell line, BHK-21, a baby hamster kidney fibroblast cell line and Vero-isolated from kidney epithelial cells extracted from African green monkey. The cytotoxicity effect of green synthesized α-Fe_2_O_3_ nanoparticle are shown in Table 4 and Figure 13.

**Table 4:** Cytotoxicity effect of green synthesized α-Fe_2_O_3_ nanoparticle on various cell lines.

Sample	Dose	Survival of cells		
		Hela	BHK-21	Vero (Non-tumoral)
In absence of solvent	25 μL	100%	100%	100%
Presence of solvent	25 μL	>95%	>95%	>95%
α-Fe_2_O_3_ nanoparticles	25 μL (30 mg/mL)	<5%	<5%	10–20%

From the Table and Figure, it is elucidated that without solvent (deionized water) 100% of the cell lines can survive without any damage. But in presence of DI water about 5% of the cell was damaged after 48 h of intubation. However, the green synthesized α-Fe_2_O_3_ nanoparticles showed a high toxicity towards the Hela and BHK-21 cell lines and it is reported that only 5% of the cell lines were survived after 48 h. Moreover, in the case of Vero cell, about 10–20% of the cell were survived for the same doses of nanoparticle. In this study, higher toxicity was observed because of the utilization of higher doses of nanoparticles as observed by other study also (Kamaraj et al., 2019). These types of results are observed because the increasing concentration of nanoparticles might cause excessive ROS-mediated oxidative stress to the cell leading to the DNA damage.

This means that cancer cells may be more sensitive to ROS challenge than normal cells, thus it may be possible to target cancer cells by ROS-mediated mechanisms (Mitra et al., 2019). As iron-based nanoparticles can serve as a strong reactive oxygen species (ROS) inducer therefore an effective amount can selectively kill tumor cells and inhibit the growth of the tumor cells. However, Hela cells are derived from cervical cancer cells therefore such types of nanoparticles could be used for cancer treatment as it has been reported that cancer cells have higher ROS levels as wells as more oxidative DNA damage than normal cells in the same tissues.

## 4 Conclusions

The iron oxide nanoparticle was synthesized effectively through a green synthesis route by using the leaf extract of *Carica papaya* plant. The as synthesized α-Fe_2_O_3_ nanoparticles showed efficient degradation ability against remazol yellow RR dye in the presence of sunlight and about 77% of the dye was degraded within 6 h for 0.8 mg/L of dosage. Synthesized NPs exhibited moderate antibacterial efficiency on specific gram-negative and gram-positive bacterial strains. Although nanoparticles showed toxicity at high doses but it showed excellent activity (almost 95% of cells destroyed) against BHK-21 and HELA cell line indicating that they could be a promising alternative for damaging tumor cells at optimum doses. However, a detailed study is necessary to find out the exact doses and reaction conditions for utilizing the nanoparticles for these purposes.

## Declarations

### Author contribution statement

Shakhawat H. Bhuiyan: Performed the experiments; Wrote the paper.

Muhammed Y. Miah, Otun Saha, Mizanur Rahaman: Analyzed and interpreted the data; Contributed reagents, materials, analysis tools or data; Wrote the paper.

Shujit C. Paul: Conceived and designed the experiments; Performed the experiments; Analyzed and interpreted the data; Wrote the paper.

Tutun D. Aka: Conceived and designed the experiments; Analyzed and interpreted the data; Wrote the paper.

Jahidul I. Sharif, Ommay Habiba: Analyzed and interpreted the data; Wrote the paper.

Ashaduzzaman: Conceived and designed the experiments; Analyzed and interpreted the data; Contributed reagents, materials, analysis tools or data; Wrote the paper.

### Funding statement

This research did not receive any specific grant from funding agencies in the public, commercial, or not-for-profit sectors.

### Competing interest statement

The authors declare no conflict of interest.

### Additional information

No additional information is available for this paper.
